# Premature Deaths

**Published:** 1885-03

**Authors:** 


### PREMATURE DEATHS.

Strong men lose their lives by imprudent acts, while the weak, compelled to take care of themselves, often live to old age. Few men live as long as they should, because few abstain from violating some law of health. The late Dr. Mariom Sims, the founder of the Woman's Hospital in New York, said that most men die prematurely even when they die of old age.

Among these premature deaths he mentions that of Peter Cooper, who imprudently exposed himself at the age of ninety-three, took cold and died of pneumonia. Capt. Labouche, who died a few years ago in New York at the age of one hundred and eleven, also died prematurely from a cold caused by imprudent exposure.

Dr. Sims says that his own father died prematurely at the age of seventy-eight, because he did what he ought not to have done. One hot day in July, he rode thirty miles in the saddle. Having stabled his horse, he began chopping wood.

Suddenly the axe dropped from his hands, and he was paralyzed. The long ride in the sun had over-heated and fatigued his body. The violent chopping overtaxed heart and lungs, and threw the blood too forcibly to the brain. A blood-vessel in the brain gave way, letting out the blood, which, forming a clot, produced paralysis.

"As all this occurred as the result of an imprudent and unnecessary act," says Dr. Sims, "I am justified in saying that father died prematurely at the age of seventy-eight; for I am sure that without this he would have lived to be ninety-five, as his grandfather did before him."

The strength of the strong is often their weakness, while the feebleness of the weak is their strength.

